# Confirming the Suitability of a Gentamicin Dosing Strategy in Neonates Using the Population Pharmacokinetic Approach with Truncated Sampling Duration

**DOI:** 10.3390/children11080898

**Published:** 2024-07-26

**Authors:** Bonifasius Siyuka Singu, Roger Karel Verbeeck, Clarissa Hildegard Pieper, Ene I. Ette

**Affiliations:** 1 School of Pharmacy, Faculty of Health Sciences & Veterinary Medicine, University of Namibia, Windhoek Private Bag 13301, Namibia; rkverbeeck@hotmail.com (R.K.V.); ene_ette@anoixiscorp.com (E.I.E.); 2 Neonatal Unit, Windhoek Central Hospital, Windhoek Private Bag 13198, Namibia; c.pieper@iway.na

**Keywords:** dosage regimen, gentamicin, neonates, population pharmacokinetics, sepsis

## Abstract

(1) Background: Gentamicin is known to be nephrotoxic and ototoxic. Although gentamicin dosage guidelines have been established for preterm and term neonates, reports do show attainment of recommended peak concentrations but toxic gentamicin concentrations are common in this age group. (2) Methods: This was a prospective, observational study conducted in Namibia with 52 neonates. A dose of 5 mg/kg gentamicin was administered over 3–5 s every 24 h in combination with benzylpenicillin 100,000 IU/kg/12 h or ampicillin 50 mg/kg/8 h. Two blood samples were collected from each participant using a truncated pharmacokinetic sampling schedule. (3) Results: The one-compartment linear pharmacokinetic model best described the data. Birthweight, postnatal age, and white blood cell count were predictive of clearance (CL), while birthweight was predictive of volume (V). For the typical neonate (median weight 1.57 kg, median postnatal age 4 days (0.011 years), median log-transformed WBC of 2.39), predicted CL and V were 0.069 L/h and 0.417 L, respectively—similar to literature values. Simulated gentamicin concentrations varied with respect to postnatal age and bodyweight. (4) Conclusions: A 5 mg/kg/24 h dosage regimen yielded simulated gentamicin concentrations with respect to age and birthweight similar to those previously reported in the literature to be safe and efficacious, confirming its appropriateness.

## 1. Introduction

Drug dose selection in neonates presents a challenge mainly due to a lack of clinical pharmacokinetic and pharmacodynamic data in this age group (because of ethical restrictions on dosing studies in pediatrics) to inform dosage guidelines [1]. As a result, many neonate dosage guidelines are produced from scaling available data from studies on adults and animals with the assumption that the physiology and biochemistry of newborns are geometrically similar to adults, overlooking the influence of tissue and organ development on the rates of metabolism and/or clearance of drugs and therefore their safety and efficacy at different life stages [2,3]. Linear weight-based (body weight (BW) or body surface area (BSA)) dosing strategies that do not consider ontogeny are inappropriate because BW or BSA do not provide a complete presentation of organ function in the neonatal population [3,4]. Due to a lack of prospective studies or randomized clinical trials, dosing guidelines are based on dosing recommendations informed by clinical experience and off-label use, which has devolved into a lack of consensus in hospital treatment guidelines [5,6,7]. The determination of safe and efficacious dosage regimens in children should be achieved by performing population pharmacokinetic (PPK) as well as safety and efficacy studies [3].

Gentamicin is known to be nephrotoxic and ototoxic [8,9]. Therapeutic peak gentamicin serum concentrations are considered to be 4–12 mg/L, whereas trough levels should be less than 2.0 mg/L [10,11,12]. Although gentamicin dosage guidelines have been established for preterm and term neonates, reports show that attainment of recommended peak concentrations [13,14,15] as well as toxic gentamicin concentrations are common in this age group [13,14,16,17,18]. Gentamicin PPK studies in neonates have reported the use of models that all include BW to predict clearance (CL) and volume of distribution (V), with resulting recommendations having gestational age (GA), postnatal age (PNA), BW, and serum creatinine (SCr) considered in dosage determination [13,15,19,20]. The influence of sepsis on the PK of gentamicin has been characterized and reported in the literature [21,22]. However, there is still a gap in knowledge about how white blood cells affect the PPK of gentamicin [23,24,25].

The purpose of this study was to characterize the PPK of gentamicin, including predictive covariates in neonates receiving gentamicin therapy against sepsis using a truncated sampling design, and to determine whether the 5 mg/kg/24 h dosage regimen achieves target therapeutic concentrations in this population.

## 2. Methods

### 2.1. Study Design and Setting

This was a prospective and non-randomized observational study carried out at the Neonatal Unit of the Maternity Ward, Windhoek Central Hospital, Windhoek, Namibia. The study was approved by the Human Research Ethics Committee of the University of Namibia (ethical clearance reference number: H-G/431/2017) and approved by the Research Ethics Committee of the Ministry of Health and Social Services (Approval number: 17/3/3 BSS). The study was conducted according to the Helsinki Declaration on ethical principles for medical research involving human subjects.

### 2.2. Participants

Neonates admitted to the Neonatal Unit and prescribed gentamicin by the resident doctors for treatment of suspected or confirmed sepsis were recruited into the study after obtaining informed and written consent from their mothers. Information on participants’ medical history was obtained from patient charts

Neonates were suspected to have sepsis if they displayed clinical symptoms such as a spike in temperature, tachycardia, lethargy, and increased respiratory rate. Elevated inflammatory markers (C-reactive protein (CRP) and WBC) were used to confirm sepsis. The sensitivity of confirming sepsis using CRP and WBC is 90.3% [26]. However, no information was available on the severity of sepsis in study participants, and it was not considered to have adversely affected the investigation since it was not part of the study objectives. Neonates diagnosed with anemia and congenital anomalies were excluded from the study.

### 2.3. Drug Administration and Pharmacokinetic Sampling

Gentamicin was administered as a 5 mg/kg intravenous bolus dose via a cannula over 3–5 s q24h by a ward nurse, and it was combined with benzylpenicillin 100,000 IU/kg q12h or ampicillin 50 mg/kg q8h. Thereafter, two blood samples were collected by venipuncture from each participant using the informative PK profile randomized (block) sampling design for a drug exhibiting monoexponential pharmacokinetics [27], in which each blood sample was taken at a time falling in one of the following sampling blocks: 5–8 min (0.08–0.14 h) with 40 samples, 8–250 min (0.14–4.2 h) with 20 samples, or 250–450 min (4.2–7.5 h) with 40 samples. Pharmacokinetic sampling was performed in real-time.

The sampling duration was truncated to 7.5 h for logistic considerations (i.e., the need for minimal PK blood sample collection, collecting PK samples over a long duration, and collecting PK samples as, at, and when scheduled). We hypothesized that this truncated sampling scheme would yield efficient gentamicin PK parameter estimates using the informative PK profile (block) randomized design [27], which took into account knowledge of gentamicin elimination half-life of 5.10, 5.40, and 5.40 h in neonates reported by Vervelde et al. [28], Rocha et al. [29], and Hayani et al. [30], respectively. It was expected that the location of PK samples at and beyond the average of the reported elimination half-life by the above authors should yield an efficient estimate of CL and PK samples before the elimination half-life would yield efficient estimates of V [27].

Blood samples were collected into sterile 500 µL serum separating tubes (SST), centrifuged, and the serum stored and frozen in Eppendorf tubes at −20 °C until analyzed. Serum creatinine concentrations were measured by the kinetic alkaline pic rate Jaffe method using the Cobas^®^ 6000 analyzer (Roche Diagnostics, Indianapolis, IN, USA). Gentamicin concentrations were determined using the Indiko Plus™ autoanalyzer (Thermo Fisher Scientific Inc., Pleasanton, CA, USA). The manufacturer-stated lower limit of quantification for gentamicin was 0.3 µg/mL.

### 2.4. Data Analysis

#### 2.4.1. General Procedure

A structured approach to PPK analysis [31] was used to develop a predictive gentamicin PPK model in neonates. The process involved data structure revelation, identification of the base PPK model, exploratory graphical analysis for selection of covariates for testing NONMEM for the development of the full/final PPK model via the forward stepping model building approach, and determination of the appropriateness of the model for simulating gentamicin concentrations via the prediction corrected visual predictive check (pcVPC) approach.

#### 2.4.2. Analysis Software

NONMEM 7.4.1 (ICON Plc., Ellicott City, MD, USA) and Pirana version 3.6.2 (Pirana Software and Consulting BV, De Alerdink 18 Denekamp, 7591 DZ Netherlands) were used for the PPK analysis. Xpose package in R Software version 4.3.0 (the R Foundation for Statistical Computing, Vienna, Austria) was used for graphical and exploratory analysis of model outputs.

#### 2.4.3. Structural Model Identification

One- and two-compartment linear PK models were tested in NONMEM using the first-order conditional with interaction estimation method to identify the structural model that best described the data. Model identification and selection were based on standard model goodness-of-fit diagnostic plots and other goodness-of-fit criteria, such as the log-likelihood difference (LLD), residual error variance, and intersubject variability. The parameters were assumed to be log-normally distributed. The concentration data were log-transformed, and the error model was appropriately modified to the logarithm scale. This was preferred because concentrations of gentamicin spanned several logs. The likelihood ratio test was employed as the main criterion to compare successive models by using the model objective function value (OFV), which is equal to −2× log-likelihood of the data. The difference in the OFV of 5.99 from the comparison of two models with two degrees of freedom at *p* of <0.05 was taken to be significant.

#### 2.4.4. Covariate Analysis

For covariate modeling to develop the PPK model, the a priori-specified level of significance required for inclusion and retention of a covariate in the nonlinear mixed effect model was α = 0.05, as assessed by the asymptotically χ^2^ distributed likelihood ratio test. A log-likelihood difference (LLD) of 3.84 is required for a one-degree-of-freedom change. For a two- or three-degree of freedom change, the required LLD is 5.99 and 7.815, respectively.

Comparison of the actual change in OFV to the critical value determined whether the more complex model was preferred over the simpler model. A more complex model was accepted if improvement in model diagnostics and parameter estimates were observed. For non-nested models, the change in OFV was used as a relative measure of goodness-of-fit just as an Akaike Information Criterion (AIC) value would be used.

The effects of covariates on PPK parameters were tested using the model formulations described below. The relationship between the *P_avg,i_*_,_ the population prediction for the *i*th subject, and a continuous covariate was tested in the following form:

(1) Pavg,i=θk∗(Zik/ZRef)θeff,k

where $\theta_{k}$ and $\theta_{eff,k}$ are fixed-effect parameters, $Z_{ik}$ is the value of the *k*th covariate for the *i*th subject, and *Z_Ref_* is a reference value for the covariate used in the PPK model. Two model formulations were used to test for the PNA effect on CL—one used the allometric covariate model formulation in Equation (1), and the other used a logistic function given below.

(2) $$P_{avg,i}=\theta_{k}*\left(\frac{z_{ik}^{\theta_{eff1,k}}}{Z_{ik}^{\theta_{eff1,k}}+\theta_{eff2,k}^{\theta_{eff1,k}}}\right)$$

where $\theta_{eff1,k}$ and $\theta_{eff2,k}$ are fixed-effect parameters and $Z_{ik}$ is the value of the *k*th covariate for the *i*th subject.

Once the fixed and random effects parameters of the population pharmacokinetic model were estimated, the PPK model was developed using the forward-stepping approach while maintaining the principle of parsimony. Population PK parameters appropriate to the model being fitted were estimated and reported. Birthweight, GA, PNA, white blood cell count (WBC), and the reciprocal of serum creatinine (1/SCr) were tested for inclusion into the population PPK model.

#### 2.4.5. Shrinkage and Reliability of Parameter Estimates

Shrinkage of empiric Bayesian parameter estimates for the base and final PPK models were obtained directly from NONMEM outputs. The final PPK model developed was subjected to bootstrapping to determine the reliability of PPK model parameter estimates [32]. The bootstrap was performed in Perl Speaks NONMEM (PSN, version 4.8.0).

### 2.5. Predictive Performance

The model was validated to determine its appropriateness for its intended purpose [32]—the simulation of concentrations to determine if the dosage regimen (5 mg/kg q24h) for gentamicin used in the study population was optimal for the population. Validation was performed using the pcVPC approach in PSN (version 4.8.0). 500 simulated datasets were generated using the final PPK model. The pcVPC was performed by plotting the observed plasma concentration–time data with the corresponding 5th, 50th, and 95th percentiles of the model-based predictions. For the appropriateness of the model to be established, approximately 90% of the observed values should fall between the 5th and 95th percentiles (i.e., the 90% prediction interval) of model predictions.

## 3. Results

### 3.1. Patient Characteristics

A summary of the demographics of neonates who participated in the investigation is in Table 1. Of the neonates who participated in the study, 43 were preterm with gestational age < 37 weeks, and the rest were ≥37 weeks.

### 3.2. Pharmacokinetic Sampling

Of the fifty-two neonates, fifty were PK evaluable, and each of them provided two PK samples. Therefore, 100 samples were obtained: 8% from the 0.08–0.14 h PK sampling block, 68% from the 0.14–4.2 h PK sampling block, and 24% from the 4.2 h and higher block. A total of 38% of samples were taken less than 1.0 h after the dose, and the median (range) time between the first and the second sample was 3.17 h (0.17 to 6.5 h).

### 3.3. Base Model Identification

**Figure 1:**
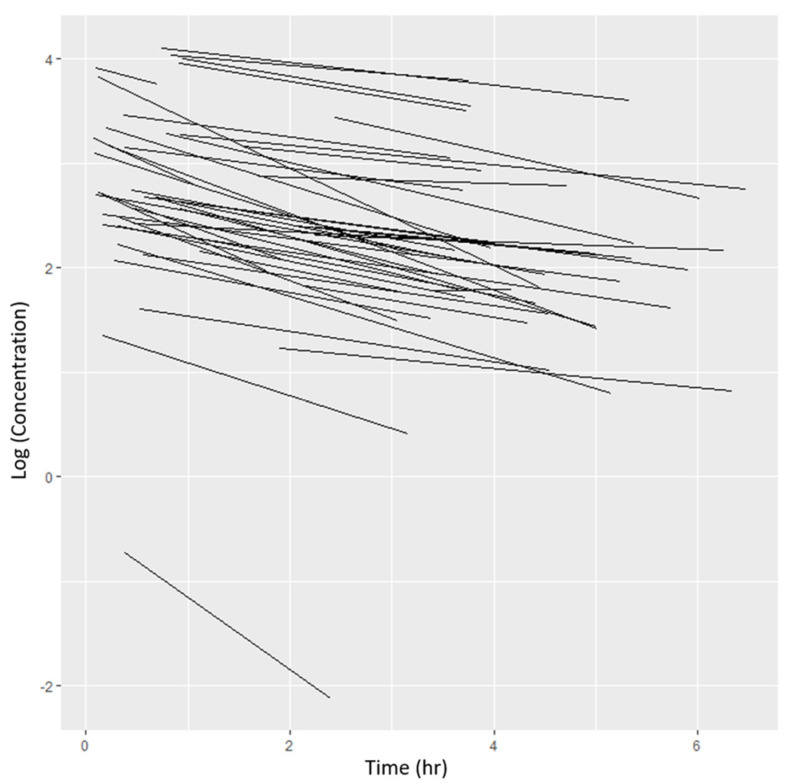
A scatter plot of gentamicin concentration-time plot in neonates.

A log-transformed concentration–time plot (Figure 1) suggested that the one-compartment PK model could best describe the data. Given the truncated PK sampling scheme, the PK sampling duration was not as long as those reported in the literature, where a bi-exponential model was used to characterize gentamicin PK [33,34]. However, comparing the results of the one- and two-compartment linear PK models’ fit to the data showed that the one-compartment model (OFV: 15.076) described the data better than the two-compartment model (OFV: 7602.386).

**Figure 2:**
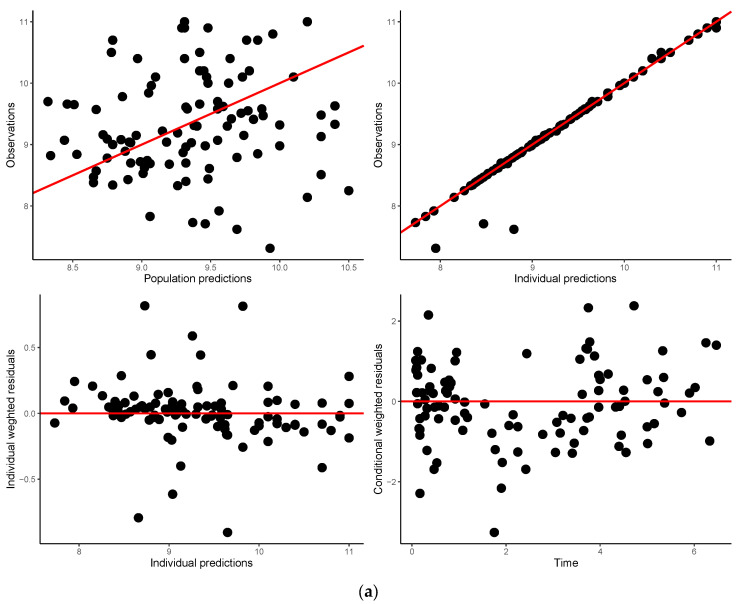

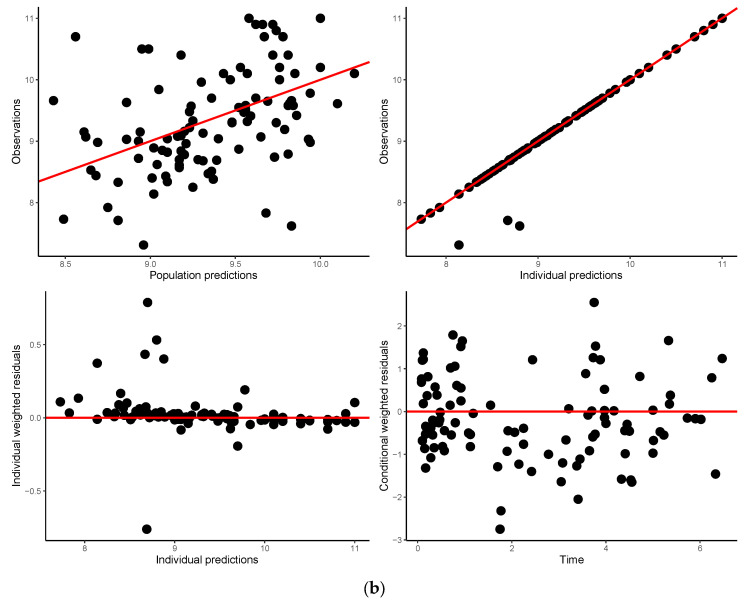
(**a**) Goodness-fit-plots from the base model. (**b**) Goodness-fit-plots from final model.

The conditional weighted residual (CWRES) versus time plot did not show discernable trends in the residuals. The plot for CWRES versus population predictions shows some bias in the lower concentrations, which could be corrected by introducing covariates into the model (Figure 2a). This was also the case for the observed versus population-predicted concentrations plot (Figure 2a). Estimates of CL and V with the associated intersubject variability from the base model were 0.0835 L/h and 0.469 L, respectively, and the associated intersubject variability in CL and V were 94.8% and 91.2%, respectively.

### 3.4. Population Pharmacokinetic Model Development

Graphical analysis showed that interindividual variability in CL (CL (Eta CL)) and interindividual variability in (Eta V) appeared to be related to PNA, while the relationship between these parameters and birthweight was not clearly discernable. Eta CL was slightly related to log-transformed *WBC*, and the 1/SCr showed no apparent relationship with these parameters. To reduce collinearity in the covariate vector, the covariate that approximated kidney function was taken to be the reciprocal of serum creatinine (1/SCr). The covariates that appeared to be related to the unexplained variability in CL (η_CL_) and V (η_V_) were included in the models tested for CL and V in developing the PPK model. Birthweight, PNA, and WBC were found to influence CL, while V was influenced by birthweight. Table 2 is the model run log summarizing the results of covariate testing and comparison of covariate models to arrive at the final PPK model. The final CL model was given by the following:

(3) CLi=CLTV,REF∗WBCWBCREFCLWBC∗WTWTREFCLWT∗FMAT

where *CL_i_* is the *CL* for the *i*th subject and *CL_TV,REF_* is the clearance for the typical (average or reference) subject, while WBC is the WBC for the *i*th subject and *WBC_REF_* for the reference subject. WBC was log-transformed, WT is birthweight, *CL_WBC_,* and *CL_WT_* are regression coefficients for WBC and WT, respectively, on CL. *FMAT* is the logistic age function (i.e., *PNA^GMMA^/(PNA^GAMMA^+PNA_50_^GAMMA^*)) that accounts for the rapid changes in gentamicin CL in the first hours of life (first day of life defined as day 1); *GAMMA* is the steepness parameter, and *PNA_50_* is the estimated PNA for CL to reach fifty percent of maturity, given the dataset. The model for V was as follows:

(4) Vi=VTV,REF·WTWTREFVWT

where *V_i_* is the *i*th subject volume of distribution, *V_TV,REF_* is the volume of distribution for the typical subject, *WT* is the weight for the *i*th subject, and *WT_REF_* is the weight for the typical subject. *V_WT_* is the regression coefficient of weight on V. Thus, weight was predictive of V. Postnatal age, *WBC*, and weight were predictive of CL. Although the confidence interval for weight included 1.0, it was retained in the CL model for a biological reason (i.e., dosing based on allometry, Table 3).

Goodness-of-fit plots generated with the final PPK model, given the data, show evenly scattered points around the line of identity in the plot of observations vs. population predictions and the plot of observations vs. individual predictions (Figure 2b). The CWRES versus time plot shows that the final model adequately characterized the data (Figure 2b). The CWRES versus time plot shows that most of the bias in predicting the low concentrations was eliminated in the final model (Figure 2b).

The final model yielded an OFV of −22.151; the difference in OFV was −37.227 when compared with the base model (OFV = 15.076), and the BSV on CL (i.e., 0.31%) and V (i.e., 70.8%) reduced by 67.3% and 22.4%, respectively, when compared with corresponding values obtained with the base model. Shrinkage for the final model was 3.19% for ηCL and 3.18% for ηV. The fixed effects parameters were relatively precise (Table 3). For the typical (average) neonate in the PK dataset (median weight 1.57 kg, median postnatal age of 4 days (0.011 years), median log-transformed WBC of 2.39), the predicted CL and V are 0.069 L/h and 0.417 L, respectively. The elimination half-life of 4.2 h was obtained for the typical subject in this study from the relationship of CL and V.

The estimate of the variability in CL was infinitely small. Estimates of intersubject variability in V and PNA_50_ were relatively precise. The two components of the residual error were estimated without fixing the additive component to zero. The model could not minimize successfully without it.

A combination residual error model was examined for adequacy in describing the concentration data. Subsequently, two combination error models for residual variability for log(concentration) ≤7.725 and >7.725 (i.e., 2264.25 ng/mL) were investigated, and this resulted in a significant decrease in the OFV by 10. The 2264.25 ng/mL threshold, which was selected based on sensitivity analysis, resulted in the highest decrease in the objective function. The threshold had no clinical or therapeutic implications. This approach to residual error model characterization was previously reported by Hussein et al. [35].

### 3.5. Model Validation

**Figure 3:**
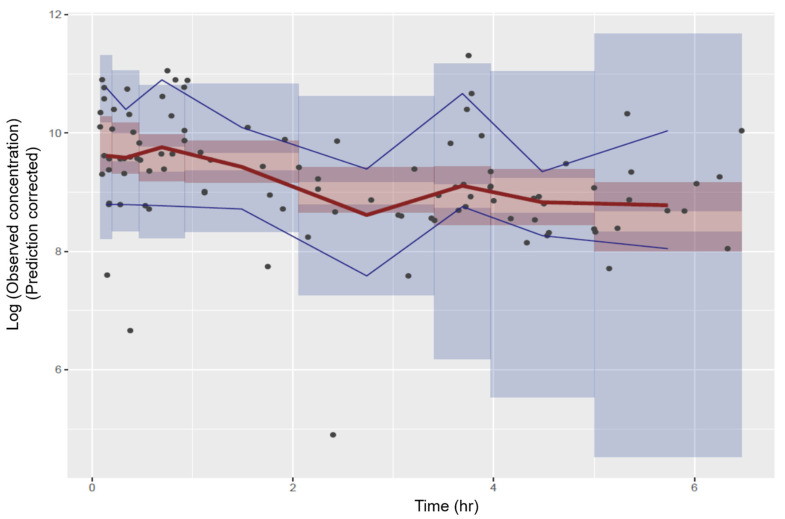
Prediction-corrected visual predictive check. Note: the black dots are the gentamicin observed concentrations; the red bold line is the 50th percentile prediction; the lower and upper blue lines are the 5th and 95th prediction percentiles; and the bands about the lines are the 95% prediction intervals about the percentiles.

The prediction-corrected visual predictive check performance plot showed that the model appropriately characterized the data and was, therefore, generalizable. The 5th, 50th, and 95th percentile predictions were within the respective 95% prediction intervals (Figure 3). The model’s generalizability meant that it was suited for its intended purpose—the simulation of gentamicin concentrations to confirm the suitability of the 5 mg/kg/24 h dosage regimen for the study population.

### 3.6. Model Application

Considering birthweight, the median values for C_min_ and C_max_ from the simulated gentamicin exposures were 1.18–1.26 mg/L and 6.83–6.96 mg/L, respectively. Although the values were not very different, neonates with a higher PNA seemed to have lower C_min_ and C_max_ concentrations, while those with a higher birthweight had slightly higher C_min_ and C_max_ concentrations. Median concentrations above the target range were between 12.40 and 12.80 mg/L with respect to postnatal age and 12.40 and 12.70 mg/L for birthweight, with the highest 95th percentile of 14.66 mg/L and 14.90 mg/L, respectively. Simulated gentamicin concentration distributions with PNA and birthweight are summarized in Table 4.

## 4. Discussion

### 4.1. Gentamicin Population Pharmacokinetics

To our knowledge, this is the first study to describe the population PK of gentamicin in neonates with suspected or confirmed sepsis using a truncated PK sampling duration. In addition to identifying clinically influential covariates, an important objective of this study was to confirm whether the dosage regimen employed at the neonatal intensive care unit that served as our study site achieves the recommended target therapeutic concentrations.

Gentamicin disposition pharmacokinetics has been described in the literature with either monoexponential [13,20,22] or biexponential PK models [33,36,37]. The one-compartment PK model best described our data and yielded efficient PK parameter estimates. The logistic age model of PNA used in this study accounted for the rapid changes in gentamicin CL in the first hours of life (first day of life defined as day 1) [33]. This PNA age function has been previously used by Germovsek et al. [33] to characterize the effect of postmenstrual age and PNA on gentamicin CL in a neonatal population with demographics similar to the one in this study. In this study, it performed better than the PNA allometric age model. The large variability associated with PNA_50_ results from not having an adequate spread of samples in the region of the profile that contained information on the fixed effect parameter. It is worth noting that the parameter estimate was estimated with good precision and negligible bias, as indicated by a minimal shrinkage of 1.26%.

The infinitely small variability in CL obtained was because the spread of the PK samples in the region of the PK profile containing information for CL estimation was inadequate for its estimation. For a given sample size, mean structural model parameters are usually better estimated than the associated variability [27]. The estimate was not fixed because it was important to estimate the covariance between CL and V at the individual subject level, which was essential for the efficient simulation of gentamicin concentrations.

Using the final model CL estimate, the CL predicted for the average (typical) neonate in our study was similar to those predicted with the models reported by Sherwin et al. [34] and Thomson et al. [20] using the typical neonate demographics in this study. Although the CL reported by Sherwin et al. [34] for neonates with sepsis was 0.085 L/hr, assuming an average body weight of 2 kg and a postnatal age of 4.4 weeks in that study [34], using the body weight and postnatal age for the typical patient in this study with the CL equation reported by Sherwin et al. [34] yielded a predicted CL of 0.06 L/hr, which is similar to what was estimated for the average patient in this study. Similarly, using the CL equation reported for neonates by Thomson et al. [20] with the weight and postnatal age of the average neonate in this study yielded a CL of 0.069 L/hr. Thus, the CL estimated with PK data obtained with the truncated sampling strategy used in this study is similar to those reported by Sherwin et al. [34] for neonates with sepsis and Thomson et al. [20] for neonates. These authors used the one-compartment linear PK model to analyse their PK data. Using the two-compartment linear PK model to analyse gentamicin PK data from premature newborns with demographics [PNA (5.49 ± 5.41 days), gestational age (32.19 ± 2.97 weeks), and body weight (1.68 ± 0.63 kg)] similar to those of the typical neonate in our dataset, Garcia et al. [38] reported a CL estimate of 0.0674 L/h—a value similar to the CL value of 0.069 L/h for the typical neonate in our dataset.

In addition, the CL for the typical neonate in our study is similar to a value of 0.08 L/h obtained by applying the 1.57 kg weight of the typical preterm neonate in our study to the 0.85 L/min/kg reported by Rocha et al. [29], who studied neonates with gestational age (31.3 ± 4.1 weeks) similar to ours. These authors reported a V of 0.4 L/kg, which is equivalent to a V of 0.628 L for the typical neonate in our study who weighed 1.57 kg—a value similar to the estimated V of 0.417 L in our study for a typical neonate who weighed 1.57 kg. The average elimination half-life of 5.4 h reported by Rocha et al. [29] is similar to the elimination half-life of 4.2 h obtained for the typical subject in this study from the relationship of CL and V.

The similarity of the CL estimate from this study to those reported by Sherwin et al. [34], Thomson et al. [20], and Rocha et al. [29], who used the one-compartment model, and Garcia et al. [38], who used the two-compartment model to describe the PK of gentamicin, is because CL is a model-independent parameter. The agreement of our CL estimate with literature-reported values [20,29,34,39] explains why the Cmin and Cmax values predicted with our model agree with values reported in the literature [20,31,32]. This indicates that the PK profile (block) randomized PK design with truncated PK sampling was adequate for estimating CL, the most important PK parameter for multiple dosing and dosage adjustment.

The similarity in CL and V estimates for the typical neonate in our study to that reported by Rocha et al. [29], assuming the 1.57 kg weight of neonate in our study, explains the similarity of gentamicin elimination half-life in our study and theirs. Thus, an efficient estimate of V was obtained despite the logistical challenges encountered with sample collection in this study. This is because enough samples were located in the 0.08 to 0.14 h and 0.14 to 4.2 h sampling blocks to efficiently estimate V with its associated intersubject variability. A sample size of ≥50 coupled with PK profile (block) randomized sampling time design has been shown to be sufficient for efficient PPK parameter estimation for drugs exhibiting one-compartment linear PK [27], and we had 50 PK-evaluable neonates in our study.

Efficient PPK parameter estimates obtained with the truncated PK sampling duration are attributed to the adequate allocation of PK samples to regions for the PK profile for the estimation CL and V—a consequence of using the PK profile (block) randomized sampling design [27]. The literature has discussed maximum PK information about model parameters at certain regions of the plasma/serum concentration-time profile [40].

The final model characterizing CL included birth weight, age, and white blood cell count, while that for V included WT. Several studies have reported that body weight and age significantly influence PK gentamicin in neonates [19,20,41,42,43].

Our study is the first to report the effect of WBC on gentamicin CL in neonates with sepsis. In addition to the nephrotoxicity of gentamicin, sepsis is characterized by inflammatory processes (WBC being a marker), which lead to acute kidney injury and chronic renal dysfunction with a significant decline in eGFR [41,43,44]. This is why WBC predicts gentamicin CL, hence its PK, in neonates with sepsis. Sherwin et al. [34] previously reported sepsis to be a predictor of gentamicin PK, although they used C-reactive protein, and not WBC, as the marker for sepsis. Even though Sherwin et al. [34] used C-reactive protein (CRP) as the marker of sepsis in their report, other studies have reported that WBC count was significantly associated with culture-proven neonatal sepsis [26,45,46,47]. WBC has been shown to have a neonatal sepsis diagnostic performance similar to CRP [26]. This informed the use of WBC as the marker of sepsis in this study. A study with a larger sample is needed to further confirm the importance of WBC as a predictor of gentamicin PK.

Repeated blood sampling may increase the need for blood transfusions since it is associated with the depletion of circulating blood volume in neonates [48], hence the limitation of the number of samples to two per neonate. Physiologic changes such as blood pressure alteration during blood drawing have been incriminated in the pathogenesis of conditions such as intravascular hemorrhage [49]. Pediatric investigations, therefore, must afford an acceptable compromise between patient considerations and the quality and quantity of science [50]. Experimental methods that minimize risk and discomfort to the patient while meeting rigorous standards for accuracy and precision in determining pharmacokinetic parameters should be used. Consequently, the study was designed to maximize the amount of information available from a relatively limited number of observations per patient in the neonate population. These considerations led to using a truncated PK sampling scheme with a two-sample design [51] based on the informative PK profile (block) randomized design.

Generally, sample times can be located in regions of the PK profile to improve the information content of the available concentration–time data. The maximization of PK information about model parameters at certain key time points [40,52] or regions of plasma concentration-time profile [53] has been reported in the literature. The results of our investigation indicate that enough samples were already located in the critical regions of the PK profile, per the informative PK profile (block) randomized design, for efficient estimation of the PPK parameters with the truncated PK sampling scheme.

### 4.2. Application

To demonstrate the applicability of the developed gentamicin PPK model, the validated PPK model was used to simulate concentrations of gentamicin, assuming the 5 mg/kg/24 h dosage regimen. The simulated gentamicin concentration distributions with respect to age and birthweight from the 5 mg/kg/24 h dosage regimen in this population of neonates indicate that the dosage regimen yields gentamicin exposures that are reported to be safe and efficacious [54,55]. A 3–5 mg/kg gentamicin dose at intervals of either 24 h, 36 h, or 48 h is recommended against neonatal sepsis by dosing guidelines for neonates such as the British National Formulary for Children (BNF-c), Neofax^®^, and the Neonatal Guidelines and Drug Doses (NGDD) [55,56,57,58]. Both the Neofax^®^ and NGDD recommend dose and dosing intervals according to GA and PNA, whereas the BNF-c only uses PNA. Studies have supported the efficacy and safety of the 3–5 mg/kg dosing regimen with a target peak serum gentamicin concentration range of 5–12 mg/L without renal function impairment in neonates receiving the dose [48,59,60]. Renal impairment was not an issue with our study population. Our results confirm the suitability of the 5 mg/kg/24 h dosage regimen used in treating neonates with sepsis. However, the assurance of safety in neonates could be improved by implementing recently developed innovations such as the rapid point-of-care genotyping method to avoid ototoxicity due to aminoglycoside drugs [61].

### 4.3. Challenge

The challenge faced in this study was that researchers were not in control of administering gentamicin doses and the exact time at which blood samples were drawn. This was because the study was conducted at a state hospital facility with established standard treatment procedures/systems which could not accommodate our study protocol preferences. Pharmacokinetic sample collection completely depended on clinical personnel employed in the ward for blood draws. Despite the logistical challenges, the use of the PK profile (block) randomized sampling time design [27] with the truncated sampling strategy yielded efficient PPK parameter estimates, enabling the use of the validated PPK model developed for simulating gentamicin concentration for the determination of the appropriateness of the 5 mg/kg/24 h dosage regimen using peak and trough concentrations.

## 5. Conclusions

The one-compartment linear PK model best described the data in this study, with the final model CL estimate similar to those previously reported in the literature [20,29,36,37,38] and the V estimate similar to that previously reported [29]. Using a truncated PK sampling schedule in conjunction with the PK profile (block) randomized sampling design [27] in our study, a validated gentamicin PPK model was developed with efficiently estimated parameters, making the model useful for simulating concentrations of gentamicin in neonates using an extended sampling duration with multiple dosing for the characterization of gentamicin peak and trough levels.

The final model characterizing CL included birth weight, age, and WBC, while that for V included birth weight as an influential covariate. Our study is the first to report WBC as a covariate predictive of gentamicin PK in neonates with sepsis. The simulated gentamicin concentration distributions with respect to age and birthweight after a 5 mg/kg/24 h dosage regimen in this population of neonates indicated that the dosage regimen yielded concentrations reported in the literature to be safe and efficacious, confirming its appropriateness.

## Figures and Tables

**Table 1:** Demographics of the neonatal study.

Characteristic	n (%)
Females	23 (44.2)
Males	29 (55.8)
Preterm (<37 weeks GA)	43 (82.7)
Total number of neonates	52
	**Median (range)**
Birth weight (kg)	1.57 (0.90–3.92)
Gestational age (weeks)	32 (24–40)
Postnatal age (days)	4.0 (1.0–17)
Height (cm)	41 (30–53)
White blood cell count (×10^9^/L)	11.0 (1.67–37.4)
Serum creatinine (mg/dL)	0.72 (0.20–1.66)
Gentamicin dose (mg)	7.9 (4.0–17)

**Table 2:** Comparison of models.

Run Number	Description	cf	OFV	LLD	df	LRT * Significant
1	Base Model: CL_Ref_					
	V_Ref_	-	15.076			
2	CL~WT	1	9.903	−5.173	1	yes
3	CL~PNA	1	11.499	−3.577	1	no
4	CL~FMAT	1	1.802	−13.274	2	yes
5	CL~WBC	1	0.653	−14.423	1	yes
6	Cl~RSCR	1	12.543	−2.533	1	no
7	V~WBC	1	14.989	−0.087	1	no
8	V~WT	1	−7.403	−22.479	1	yes
9	CL~WBC, FMAT	4	−2.346	−4.148	1	yes
10	CL~WBC, FMAT, WT	9	−8.675	−6.329	1	yes
11	CL~WBC, FMAT, WT	10	−22.151	−13.476	1	yes
	V~WT					

cf—compared with. df—degree of freedom. OFV—objective function value. LLD—log-likelihood difference. LRT—likelihood ratio test. Note: FMAT = *PNA^GMMA^*/(*PNA^GAMMA^* + *PNA*_50_*^GAMMA^*), where GAMMA is the shape parameter and PNA_50_ is the postnatal age for CL to reach 50% maturity. Model runs 2, 3, and 5 to 8 used the allometric model formulation in Equation (1), while model run number 4 used the logistic formulation in Equation (2). * The LLD used in the LRT follows a Chi-squared distribution with critical values at the 5% significance level for one and two degrees of freedom are 3.84 and 5.991, respectively.

**Table 3:** Summary of parameter estimates from the final model.

Parameter	Original Estimate	90% Bootstrap Confidence Interval	Shrinkage (%)
CL (L/h)	0.196	0.132, 0.228	
V (L)	0.417	0.330, 0.476	
V_WT	1.76	1.39, 2.34	
CL_WT	1.30	0.558, 1.68	
CL_WBC	−0.560	−1.70, 0.375	
GAMMA	0.551	Fixed	
PNA_50_ (yr)	0.0332	Fixed	
^a^ Intersubject variability			
$\omega^{2}CL$	1.00 × 10^−5^ (0.31%)	−0.289, 0.289	3.19
$\omega^{2}CL:V$	0.00024	−0.221, 0.234	
$\omega^{2}V$	0.501 (70.8%)	0.436, 0.679	3.18
$\omega^{2}{PNA}_{50}$	6.23 (250%)	9.10, 13.4	1.26
^b^ Residual variability			
Res_ADDITIVE1_	4.96	2.99, 16.83	9.37
Res_PROPORTIONAL1_	−0.99	−1.235, −0.745	
Res_ADDITIVE2_	1.00 × 10^−5^	−0.00017, 0.00019	
Res_PROPORTIONAL2_	0.0205	−1.95, 1.96	

CL_TV_ and V_TV_ are the clearance and volume of distribution for the typical (i.e., average or reference) subject in the dataset weighing 1.57 kg; CL_WT and V_WT are the regression coefficients for birthweight on CL and V, respectively; CL_WBC is the regression coefficient for WBC on CL. GAMMA is the shape parameter, and PNA_50_ is the postnatal age for CL to reach 50% maturity. ^a^ Intersubject in CL, V, and PNA_50_ (i.e., ω^2^_CL_, ω^2^_V_, and ω^2^_PNA50_, respectively) estimated as their variances, and the numbers in parentheses are the respective intersubject expressed as percentages. ω^2^_CL:V_ is the covariance between CL and V. ^b^ Residual errors are estimated as their standard deviations. GAMMA and PNA_50_ were initially estimated and later fixed in the final model.

**Table 4:** Summary of simulated gentamicin concentration distributions with age and birthweight after a 5 mg/kg/24 h dose in neonates.

	Concentration Percentile								
	Cmin (<2 mg/L)			Cmax (5–12 mg/L)			Concentration above 12 mg/L		
	5th	50th	95th	5th	50th	95th	5th	50th	95th
**Postnatal age (days)**									
1–2	0.52	1.30	1.94	5.27	7.28	10.30	12.10	12.80	14.66
3–5	0.43	1.26	1.93	5.12	6.89	10.10	12.10	12.60	14.00
6–10	0.46	1.26	1.93	5.07	6.56	9.88	12.23	12.40	13.97
>10	0.42	1.21	1.88	5.06	6.55	9.61	12.70	12.70	12.70
**Weight (g)**									
<1000	0.42	1.18	1.88	5.09	6.96	10.10	12.14	12.40	13.62
1000–1499	0.41	1.25	1.93	5.11	6.83	10.00	12.10	12.70	14.90
1500–2499	0.45	1.26	1.91	5.13	6.90	10.20	12.10	12.70	14.00
>2500	0.53	1.40	1.94	5.23	7.22	10.20	12.10	12.70	14.60

## Data Availability

The raw data supporting the conclusions of this article will be made available by the authors on request.
